# New regulations regarding Postgraduate Medical Training in Spain: perception of the tutor's role in the Murcia Region

**DOI:** 10.1186/1472-6920-10-44

**Published:** 2010-06-14

**Authors:** Jose Galcerá-Tomás, Carmen Botella-Martínez, José Saura-Llamas, Fernando Navarro-Mateu

**Affiliations:** 1 Postgraduate Teaching Unit, University Hospital "Virgen de la Arrixaca", Ctra, Madrid -Cartagena s/n. 30120-El Palmar, Murcia, Spain; 2 Family and Community Health Teaching Unit, C/Escultor Sánchez Lozano, 5. 30007, Murcia, Spain; 3 Mental Health Multiprofessional Teaching Unit, C/Lorca, 58.,30120-El Palmar, Murcia, Spain

## Abstract

**Background:**

Recently introduced regulatory changes have expanded the Tutor role to include their primary responsibility for Postgraduate Medical Training (PMT). However, accreditation and recognition of that role has been devolved to the autonomic regions. The opinions of the RT may be relevant to future decisions;

**Methods:**

A comprehensive questionnaire, including demographic characteristics, academic and research achievement and personal views about their role, was sent to 201 RTs in the Murcia Region of Spain. The responses are described using median and interquartile ranges (IQR);

**Results:**

There were 147 replies (response rate 73%), 69% male, mean age 45 ± 7 yrs. RTs perception of the residents' initial knowledge and commitment throughout the program was 5 (IQR 4-6) and 7 (IQR 5-8), respectively. As regards their impact on the PMT program, RTs considered that their own contribution was similar to that of senior residents. RTs perception of how their role was recognised was 5 (IQR 3-6). Only 16% did not encounter difficulties in accessing specific RT training programs. Regarding the RTs view of their various duties, supervision of patient care was accorded the greatest importance (64%) while the satisfactory completion of the PMT program and supervision of day-to-day activities were also considered important (61% and 59% respectively). The main RT requirements were: a greater professional recognition (97%), protected time (95%), specific RT training programs (95%) and financial recognition (86%);

**Conclusions:**

This comprehensive study, reflecting the feelings of our RTs, provides a useful insight into the reality of their work and the findings ought to be taken into consideration in the imminent definitive regulatory document on PMT.

## Background

Postgraduate Medical Training (PMT) based on the North American Residency Model was introduced in Spain in the 1960s and '70s [1] although a regulatory framework was not established until 1984 and 1995 [2] and this also defined the role of the Residents' Tutor (RT). Formal PMT Programs were introduced in the Murcia Region in the 1970s and nowadays include over 500 residents in more than 40 specialities. All PMT Centres and Programs have been accredited by the Spanish Health Ministry and each centre has its own Teaching Committee with responsibility for compliance. Recently introduced changes have expanded the role of the RT, specifically increasing his/her responsibility in planning and actively collaborating in the acquisition of knowledge, skills and positive attitude of the residents for the purpose of guaranteeing the successful completion of the training programme in the relevant specialty. Moreover, it has been established that the RT is the one who has the primary responsibility for the Residents' teaching/learning process since he will have continuous contact and provides a structured and supportive role in whatever specialty the training process takes place [3]. This additional responsibility which the new rules place on the RT in Spain makes it difficult to compare that role with that of Tutors from other countries or indeed with that from other non-medical postgraduate training programmes [4-9]. In spite of the different peculiarities of the Tutor role, a common challenge of this position is the need to be able to deal with problems and conflicts whilst not holding a formal academic or hierarchical position [10] and/or when regulatory changes are introduced [11]. In our particular situation, the current regulations have left some very relevant and important issues unaddressed. For example, the issues of recognition of time and effort spent in this role and accreditation have been devolved to the Autonomic Communities [3] consistent with the Spanish decentralised National Health System [12]. However, the allocated period for the implementation of these additional elements by the Autonomic Communities expired in February 2009 and, although regrettable, this has allowed us time to formally reflect on the views and opinions of our Tutors, the results of which may hopefully be taken into count in their final deliberations.

Mindful of these issues, the present study was designed to identify the typical profile of our current Tutors as well as their views and perceptions in relation to their role at a time of ever-increasing demands being placed upon them.

## Methods

A questionnaire which was devised by a group of PMT experts was sent via the Chief of Studies to all 201 RTs in the Murcia region, including five Hospital and six Primary Care areas. It consisted of 72 questions which included, amongst others, demographic characteristics, number of years in professional practice and specifically, as Tutor, academic and research achievement as well as their personal views on other aspects of their role including the importance of the various Tutor functions, degree of collaboration perceived within their service, method of evaluation of the residents and their level of awareness of the regulations governing their role and function as Tutor. Other questions sought to obtain the Tutors' views of their residents' initial knowledge and commitment throughout the program as well as their own requirements for adequately fulfilling their role as Tutor and the level of importance accorded to the various Tutor functions.

A scale of 0-10 was used in some of the questions as is commonly used in Spanish education system surveys, including evaluations of Tutors in this field [13] whereas for some other variables a qualitative-type Likert scale [14] was used to facilitate the interpretation of the responses. Categorical variables are expressed as percentages and continuous variables as mean and standard deviation. The scaled 0-10 responses are represented as median, ranges and interquartile ranges. For some purposes the upper quartile was considered "high". Tutor profiles and the performance of various functions were compared depending on whether they scored their knowledge as high or otherwise by a Chi square test.

The study was approval by the Murcia Health Area 1 Ethical Committee and it complies with the 1975 Declaration of Helsinki.

## Results

**Figure 1:**
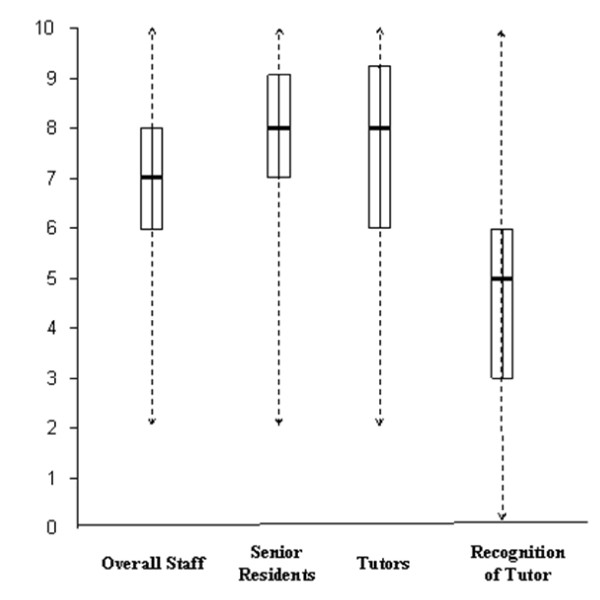
**Tutors' perception of residents' initial knowledge, commitment throughout the program, and collaboration and awareness of the PMT service as well as Tutors' knowledge of the rules**. Data expressed as median (horizontal black bars), ranges (dashed arrows) and interquartile ranges (rectangular areas)

**Figure 2:**
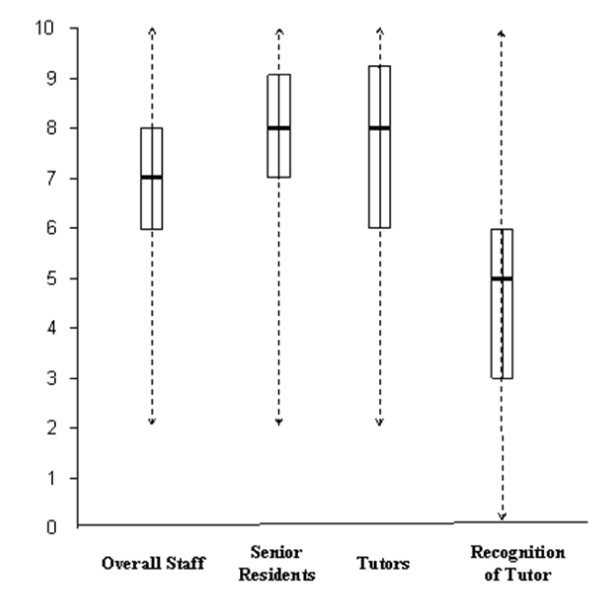
**Tutors' views of the contribution to the successful completion of PMT program by staff, senior residents and Tutors, as well as level of recognition of the Tutor role**. Data expressed as median (horizontal black bars), ranges (dashed arrows) and interquartile ranges (rectangular areas)

Of a total of 201 Tutors with responsibility for residents in the region of Murcia, 147 replied (73% - 95 Hospital and 52 Family Tutors). The Hospital Tutors were based at the five centres accredited for PMT in the region (Virgen de la Arrixaca, Santa María del Rosell, Reina Sofía, Morales Meseguer, and Rafael Méndez) whilst the Family Tutors surveyed were working in six Primary Care areas. The main demographic features and academic/research profile of the Tutors, including their view on the inclusion of a common training period in the residency programme, are shown in Table 1. The Tutors' opinion of the residents' level of initial knowledge, their commitment throughout the training program, and their own level of knowledge of the new regulations are shown in Figure 1 as is their perception of the degree of collaboration and actual awareness of the PMT program within their service. Their opinion of the contribution to the successful completion of the PMT program by various groups as well as their own views of how their role is perceived by others within the service is shown in Figure 2.

**Table 1:** Demographic features, academic/research profile and Tutors' views on a common training period.

Male	69
Age (yrs)*	45 ± 7
Time since qualification (yrs)*	20 ± 9
Time as Tutor (yrs)*	5.3 ± 5.0
Number of residents per year*	3.7 ± 3.1
Philosophical Thesis	37
Attendance at Tutor Training Programs	54
Membership of Teaching Committee	47
Publications (≥ 10)	33
In favour of a common training period	74

Data expressed as percentages in absence of other criterion

* Data expressed as Mean ± Standard Deviation

As regards the day-to-day functions across all specialties, only 48% of Tutors formally monitored the residents' log book. In contrast, 93% did plan formal clinical sessions, 81% provided a complete rotation program for their residents and 75% of Tutors had regular and structured appraisals with them. Notwithstanding these variable rates of compliances, only 72% considered that the teaching and learning objectives of the formal PMT program were adequately achieved (Table 2).

**Table 2:** Various tutor functions performed and tutors'perceptions of the degree of satisfactory completion of PMT program.

Function/Perception	%
Clinical care supervision	65
Individualised evaluation	54
Complete rotation program	81
Structured appraisals	75
Resident Annual Report	67
Planning formal clinical sessions	93
Supervision of residents' log book	48
Satisfactory completion of PMT program	72

With respect to the Tutors' perceptions of the level of importance of their various duties, supervision of clinical care was accorded the greatest importance whilst the satisfactory completion of the PMT program and supervision of day-to-day activities were also considered highly important (Table 3). With regard to the Tutors' level of knowledge of the regulations, the median result was only 6 with an IQR of 4-7 (Figure 1). Perhaps unsurprisingly, there was a significant correlation between this level and various Tutor profiles but surprisingly, with only a single Tutor function ie. the production of an Annual Resident Report (Table 4).

**Table 3:** Level of importance given to various Tutor functions.

Function	Median	IQR	High importance (%)
Clinical care supervision	8.0	(7.0 - 9.0)	64
Satisfactory completion of PMT program	7.0	(6.0 - 8.0)	61
Supervision of day-to-day activities	7.0	(5.0 - 8.0)	59
Implementation of rotation program	7.0	(5.0 - 8.0)	54
Structured appraisals	7.0	(6.0 - 8.0)	50
Formal evaluation of residents' progress	6.0	(5.0 - 8.0)	43
Planning formal teaching sessions	6.0	(5.0 - 8.0)	40
Residents' log supervision	6.0	(3.0 - 7.0)	39

Data expressed as median, interquartile ranges (IQR) and percentage of Tutors according a high level of importance to each function.

**Table 4:** Differences between Tutor profile and performance of various functions according to level of knowledge of new Tutor regulations.

Tutor profile or function	Level of knowledge (%)		
		
	High (51%)	Non-high (49%)	p value
Attendance at formal Tutor Training Programs	68	43	0.003
Membership of Teaching Committee	58	37	0.007
Philosophical Thesis	47	27	0.03
Individual resident evaluation	60	46	0.1
Residents' log-book supervision	47	45	0.8
Resident Annual Report	31	24	0.03

Other interesting findings were that 65% of Tutors considered that their own clinical commitments had a negative impact on their contribution to adequate supervision of the residents' training programme and only 16% admitted to have no difficulties in accessing formal Tutor Training Programmes. Finally, the main Tutor requirements expressed were greater professional recognition of their role (97%), more protected time for that role (95%), easier access to specific Tutor Training Programmes (95%) and some financial recognition (86%).

## Discussion and Conclusions

The role of the Tutor and his relationship with colleagues has been the subject of much discussion for many years [8,9,15-17]. Our large regional study, using a comprehensive questionnaire with a high response rate, provides a useful and important insight into the actual day-to-day work of our Tutors as well as their perceptions of their role. It primarily shows that there are discrepancies between what is expected of them as laid down in the recent regulations, especially in their level of responsibility for supervision and training of Residents, and both their fulfilment and perceptions of those core functions.

Our results showing that several core Tutor functions, including supervision of the Residents' log book, production of an Annual Residents' Report and regular structured appraisals were only carried out in no more than three-quarters of cases is of some concern. In particular, the fact that fewer than 50% of Tutors made a detailed supervision of their Residents' log book is especially surprising given the prominence of this Tutor function in the recent regulations [3], and indeed, as far back as 1995, reference to the importance of this as an assessment and evaluation tool has been made [2]. Moreover, fewer than 40% of our Tutors considered it to be highly important as an evaluation tool. This contrasts with a recent Catalonian study where 80% of Tutors considered the log book as useful [18]. However, in this study almost a quarter of Residents did not actually complete their logbook [18] and in the Madrid study by Álvarez-Sánchez at al, the majority of the Tutors questioned considered that the logbook did not reflect the elements of each speciality and, moreover, that it was not easy to complete [19]. Other notable discrepancies in how the Tutors perceived their functions as important or otherwise were the fact that although structured appraisals were performed by three-quarters of Tutors, only half considered that this core function was of high importance. Similarly, despite almost all Tutors being actively involved in the planning of formal clinical sessions, only 40% considered that this was an important Tutor function.

The discrepancies which our study has shown between current legislation regarding core Tutor functions and actual fulfilment of those functions is also a matter of concern. There are many possible reasons for these but, in our case, the fact that almost 50% of Tutors admitted to a lack of high knowledge of current legislation is likely to be a major reason. In fact, our study has shown that certain Tutor profiles, including attendance at Formal Tutor Training Programs, Membership of a Teaching Committee and possession of a Philosophical Thesis were all significantly associated with a higher level of knowledge of the regulations regarding the role of the Tutor as was the successful submission of an Annual Resident Report. This strong correlation suggests a probable causal association between at least one core Tutor function and a high level of knowledge of the regulations.

So what are the barriers to better compliance? Almost two-thirds of our Tutors considered that their clinical commitments impacted negatively on adequate supervision and the majority had difficulties in accessing formal Tutor Training Programmes. Other studies have reported similar concerns with 80-93% of Tutors identifying this as their top priority [20,21]. Similarly, a United Kingdom National survey in Anaesthesia highlighted the importance of better access to formal Tutor Training Programs [22]. But perhaps also our findings of a level of initial knowledge by our Residents which was judged to be inadequate, in addition to a level of commitment by them seen as not totally satisfactory, also reported by others [23], are also significant barriers to greater compliance with core Tutor functions.

Despite the poor recognition of the Tutor role in our survey, there was an acceptable level of collaboration and awareness of the service by other staff members. However, somewhat surprisingly, the role of the Tutor in relation to the successful completion of the PMT Program was seen to be of no greater importance than that of the Senior Residents. Given the fact that they have the primary responsibility for the residents' training, which has been granted them in legislation, this collaboration and awareness per se would appear to be insufficient in terms of provision of support and we believe that the role of the Tutor should be given greater prominence as well as looking critically at the some of the many functions assigned to him, some of which may dilute his primary role and which may adversely affect his ability to work effectively in this role. Another perceived obstacle was the lack of protected time with almost 100% of our Tutors citing this as an essential requirement, consistent with other Spanish studies [18-21]. This is likely to become even more difficult to resolve because of the recently introduced legislation on the reduction in doctors' working hours [24].

Notwithstanding the limitations of our survey, our data showing that a high level of knowledge of the regulations is associated with a greater compliance with recognised Tutor responsibilities, would suggest that the stated priorities of the Tutors surveyed, especially those of protected time, better training and greater recognition may have a favourable impact on the outcome of the residents' PMT program. This supports the case for greater effort and investment in the Tutor role to ensure that all are aware of the current rules and regulations and are familiar with the obligations which these place upon them to enable them to effectively carry out their responsibilities as PMT Tutor.

## Competing interests

The authors declare that they have no competing interests.

## Authors' contributions

JG-T conceived of the study and its design and performed the statistical analysis. CB-M participated in its design and helped to draft the manuscript and participated in the statistical analysis. JS-Ll was the major contributor to the data collected in primary care areas and actively participated in writing the manuscript. FN-M participated in designing the questionnaire and helped write the manuscript. The questionnaire was devised in the Chief of Studies Forum of Murcia Region whose members helped coordinate the responses. All authors read and approved the final manuscript.

## Authors' information

### Chiefs of Study Forum of Murcia Region (CSFMR)

José Galcerá-Tomás (Hospital Universitario Virgen de la Arrixaca), Jacinto Fernández-Pardo (Hospital Universitario Reina Sofía de Murcia), Faustino Herrero-Huerta (Hospital Morales Meseguer de Murcia), Andrés Conesa-Hernandez (Hospital Santa María del Rosell Cartagena), Magdalena Molina-Oller (Hospital Rafael Méndez de Lorca), Julio Foncuberta-Martinez (Unidad de Medicina Familiar y Comunitaria de Cartagena), Fernando Navarro-Mateu (Unidad Docente Multiprofesional de Salud Mental de Murcia). Murcia. Spain.

## Pre-publication history

The pre-publication history for this paper can be accessed here:

http://www.biomedcentral.com/1472-6920/10/44/prepub
